# An Easy Synthesis for Preparing Bio-Based Hybrid Adsorbent Useful for Fast Adsorption of Polar Pollutants

**DOI:** 10.3390/nano9050731

**Published:** 2019-05-11

**Authors:** Razieh Sadraei, Maria Cristina Paganini, Paola Calza, Giuliana Magnacca

**Affiliations:** 1 Dipartimento di Chimica, Università di Torino, Via P. Giuria 7, 10125 Torino, Italy; mariacristina.paganini@unito.it (M.C.P.); paola.calza@unito.it (P.C.); 2 NIS Interdepartmental Centre, Università di Torino, Via P. Giuria 7, 10125 Torino, Italy

**Keywords:** adsorption, crystal violet, hybrid materials, electrostatic interaction, alumina support, contaminant of emerging concern removal

## Abstract

For the first time, γ-Al_2_O_3_ and Bio-Based Substances (BBS) hybrids (**A**-BBS) were prepared through a simple electrostatic interaction occurring between alumina, used as a support, and BBS (Bio-Based Substance from composted biowastes) carrying positive and negative charges, respectively. We evaluated the optimal amount of BBS to be immobilized on the support and the stability of the resulting **A**-BBS in order to use this novel hybrid material as an adsorbent for the removal of polar pollutants. Characterization was carried out by X-Ray Diffraction (XRD) for evaluating the crystal structure of the support, Fourier transform infrared spectroscopy (FT-IR) to evidence the presence of BBS on the hybrid material, thermogravimetric analysis (TGA) to measure the thermal stability of the hybrid materials and quantify the BBS amount immobilized on the support, N_2_ adsorption at 77 K for the evaluation of the surface area and porosity of the systems, Zeta potential measurements to evaluate the effect of BBS immobilization on the surface charge of the particles and choose the substrates possibly interacting with them. Firstly, we tested the adsorption capability of three samples differently coated with BBS toward cationic species considering various adsorbate/adsorbent ratio. Crystal Violet (CV) was chosen as model pollutant to compare the performance of the hybrid materials with those of other materials described in the literature. The adsorption data were modeled by Langmuir and Freundlich adsorption isotherms. Then, we studied the adsorption capability of the developed material towards molecules with different structures; for this purpose, two contaminants of emerging concerns (carbamazepine and atenolol) were tested. The results indicate that **A**-BBS could be applied in wastewater treatment for the removal of a significant amount of polar species. In addition, a comparison with literature data concerning CV adsorption was carried out in order to evaluate the environmental impact of synthetic routes used to prepare different adsorbents.

## 1. Introduction

The removal of pollutants from urban and industrial wastewaters is one of the most important issues to be solved by modern research. In fact, water pollution has increased in the last decades by the virtue of the disposal of industrial effluents enriched with toxic species [1] which has hazardous effects on flora, fauna, and humans. Moreover, the growth of the world population results in a limitation of water supplies and scarcity of water resources, therefore the necessity of clean water has become fundamental for our society [1,2].

In the last decades, new chemicals were detected in wastewaters as a result of new industrial processes and increased consumption of pharmaceuticals and personal care products [3,4].

Among other pollutants, synthetic dyes [5] can be found in the effluent of many industries, including textile, plastic, printing, and dye manufacturing companies. In this group of contaminants, the cationic organic aromatic dyes are ones of the most present pollutants, released from industries, causing various harmful influences on aquatic bodies as well as organisms. Therefore their removal has been deeply studied in recent years [6,7].

Crystal violet (CV) belongs to this group of contaminants. It has been widely used as a dermatological agent and biological stain as well as a coloring agent for dyeing leather, silk, wool, paper, and cotton [6,7,8,9].

Several techniques have been used to remove organic pollutants and synthetic dyes from urban and industrial wastewaters such as membrane separation [2], biological [10] and electrochemical treatments [11], flocculation, liquid-liquid extraction [12], advanced oxidation processes (AOP) [13] and coagulation. Out of methods listed above, the adsorption technique [14] has attracted considerable attention because of the simple procedures needed, because no toxic substances are produced, because of ability to treat concentrated forms of the pollutants, and to reuse the spent adsorbent via regeneration. Moreover, it plays a central role in drinking water purification and wastewater treatments [3,7,15,16].

Mesoporous materials, thanks to their large and tunable porosity find potential applications in catalysis, encapsulation of proteins, filtration and separation of large molecules, membrane technology, drug delivery, dosing, sensing, among many others [17,18] Furthermore, their large specific surface area and huge number of adsorbing active sites suggest their possible application as adsorption materials [19].

In particular, alumina found various technological applications, i.e., as electrical insulator, presenting exceptionally high resistance to chemical agents, as well as giving an excellent performance as catalyst or support for many applications [20,21].

Mesoporous γ-Al_2_O_3_ has been not only widely applied in all the field mentioned above [17,21,22] but in recent years the chance to synthesize hybrid materials to enhance the useful properties of an oxide opens the way to very interesting opportunities. The literature reports that many oxides have been modified using organic moieties able to enhance the adsorption capacity of the original material. Among others, bio-based substances (BBS), extracted from composted green wastes, are very interesting macromolecules with a complex lignin-derived structure and characterized by several functional groups (they contain long aliphatic chains, aromatic rings, and several acid and basic functional groups such as carboxyl, primary and substituted amine and amide, carbonyl, hydroxyl, phenol, ether or ester [23,24]), appealing for their importance in the valorization of the organic refuses. BBS have been found to exhibit typical properties of anionic surfactants and polyelectrolytes [25,26] and have been used in the formulation of detergents, textile dyeing baths, emulsifiers, auxiliaries for soil/water remediation, flocculants, and dispersants, as binding agents and templates for ceramics manufacture, as well as for application in agriculture and animal husbandry [27]. BBS have been reported to bear chemical similarities with humic substances, and to exhibit enhanced adsorption capacity towards polar pollutants given the presence of several carboxylate and phenolic groups carrying negative charges at neutral pH, as well as photosensitizing properties [27] promoting significant mineralization of the organic carbon [24].

Hence, the aim of this study is to prepare new hybrid materials by surface immobilization of negatively charged BBS molecules on the positively charged surface of γ-Al_2_O_3_ taking advantage of electrostatic interactions occurring between them. After characterization and stability test, the materials were used as adsorbents and compared with other systems cited in the literature (reported in Table 1) to evaluate their adsorption capacity towards the cationic dye Crystal Violet (CV). Once identified the best adsorbing material for CV removal, two Contaminants of Emerging Concern (CECs) [28], namely Atenolol and Carbamazepine, were tested.

## 2. Experimental

### 2.1. Materials

γ-alumina was kindly supplied by Centro Ricerche FIAT (Orbassano (TO), Italy), the X-ray diffraction pattern confirms the expected crystalline structure of the material consistent with the reference card 01-075-0921 related to γ-Al_2_O_3_. As a simple electrostatic interaction of the support with the BBS molecules is not expected to modify the crystalline structure of the support, the hybrid sample diffractograms are not shown.

BBS were extracted from composted organic refuses (from urban public park trimming and home gardening residues) aged for more than 180 days supplied by ACEA Pinerolese Industriale (Pinerolo (TO), Italy) [23]. The extraction procedure was described elsewhere [53].

Crystal violet (CV) was purchased from Merck (Milan, Italy) and used without any further treatment to prepare solution in ultrapure MilliQ water for adsorption tests.

Carbamazepine and Atenolol were provided by Sigma-Aldrich (Milano, Italy) in analytical purity ≥99.0% and used in adsorption experiments.

All aqueous solutions for High-Performance Liquid Chromatography (HPLC) analysis were prepared using ultrapure water Millipore Milli-QTM (resistivity >18 MΩ). All chemicals were used without further purification.

### 2.2. Preparation of Hybrid Materials

The γ-Al_2_O_3_ particles were used as support for different amounts of BBS immobilized at their surface by simple electrostatic interaction occurring between the two components carrying opposite surface charges.

Hybrid materials were prepared by mixing 1 g of γ- Al_2_O_3_ in 20 mL of distilled water containing 0.1, 0.2, and 0.4 g of BBS under stirring for 24 h at 25 °C. The pH of solution was about 6.5 during the preparation. The samples were washed with 10 mL of distilled water for 10 min and every time centrifuged at 4000 rpm for 10 min. The washing solution was tested using UV-Vis spectrophotometer to evidence the presence of leached BBS molecules. The procedure was carried out several times, till the washing solution did not evidence the presence of BBS in the UV-Vis spectra. Drying process was performed in the oven at 40 °C for 24 h. Hybrid samples were named **A**-BBS0.1, **A**-BBS0.2, and **A**-BBS0.4, the reference pure alumina sample was indicated as A.

### 2.3. Characterization Methods

X-ray diffraction (XRD) analyses of alumina support was obtained using a X’Pert PRO MPD diffractometer from PANalytical (Royston, UK), equipped with Cu anode and working at 45 kV and 40 mA in a Bragg-Brentano geometry. In this study, the flat sample-holder configuration was used.

Fourier transform infrared (FTIR) spectra were recorded in transmission mode by means of a Bruker Vector 22 spectrophotometer equipped with Globar source, DTGS detector (Billerica, Massachusetts, USA), and working with 128 scans at 4 cm^−1^ resolution in the 4000–400 cm^−1^ range. Samples were dispersed in KBr (approximatively, sample: KBr weight ratio was 0.045).

Nitrogen adsorption-desorption experiments were carried out using an ASAP 2010 Micromeritics volumetric apparatus (Norcross, GA, USA). Before the measurements, the samples were outgassed at 40 °C for 24 h. Specific surface areas (SSA) were calculated using the Brunauer, Emmett and Teller (BET) method. Pore volumes (PV) and Pore Size Distribution (PSD) were determined by the Barrett, Joyner, and Halenda method [54] applied to the isotherm desorption branch.

Zeta potential measurements were performed on the instrument Zetasizer ZS90 by Malvern (Malvern, UK). 10 mg of BBS and hybrid materials were suspended in 20 mL of deionized water under constant stirring (400 rpm) for 15 min. The zeta potential measurements were performed starting from the natural pH of the suspension then decreasing it point by point by addition of 0.1 M HCl and successively increasing it with 0.1 M NaOH. A digital pH meter (Metrohm, model 827 pH lab, swiss mode, Herisau, Switzerland) was used to measure the pH of the solution.

Thermo-gravimetric analysis (TGA) was carried out using a TA Q600 (New Castle, DE, USA). Thermal analyses were performed with a heating ramp of 10 °C/min from RT to 600 °C under air in order to quantify the amount of BBS immobilized on γ-Al_2_O_3_ particles.

### 2.4. Adsorption Procedures

#### 2.4.1. Analytical Instruments

UV-Vis spectrophotometer (Varian Cary 300 Scans, Agilent, Santa Clara, CA, USA) was used to determine the adsorption of CV (maximum absorbance at 584 nm).

A Merck-Hitachi liquid chromatographer (Knauer, Berlin, Germany) equipped with Rheodyne injector L-6200 and L-6200A pumps for high-pressure gradients, L-4200 UV-Vis detector, and a LiChrocart RP-C18 column (Merck, Milano, Italy, 12.5 cm × 0.4 cm) was used to determine the concentration of atenolol and carbamazepine during the experiments. The detection wavelength was set at 224 nm for atenolol and 284 nm for carbamazepine). Isocratic elution (1 mL min^−1^ flow rate) was carried out with 60% of phosphate buffer 1 × 10^−2^ M at pH 2.8 and 40% acetonitrile and retention times were 5 min.

#### 2.4.2. Kinetic of CV Adsorption

The kinetic of the adsorption was followed contacting 10 ppm of CV with 20 mg of adsorbing hybrid materials (total volume 10 mL) at pH 6.5 (therefore, a CV:adsorbent ratio of 1:2 wt was applied to all the preliminary measurements). The mixture was stirred vigorously under isolated orbital mixing plate (rotation at 1000 rpm) keeping the temperature at 15 °C. The adsorption phenomenon was followed measuring every 10 min the absorbance of the dye in the supernatant after centrifugation (at 4000 rpm for 10 min) by means of a UV–Vis spectrophotometer. Although an equilibrium time of less than or equal to 10 min was evidenced, all the experiments were carried out leaving adsorbent and adsorptive in contact for 30 min. All the measurements (data not showed) were carried out in duplicate and the average data are reported.

#### 2.4.3. CV Adsorption Study and Model Application

The adsorption experiments performed on the hybrid absorbents were carried out at 15 °C and pH = 6.5 (natural value of the suspensions in CV) modifying the relative amount of CV and adsorbent in order to explore a wide Ce range (10 to 100 mg of dye were mixed with 20 mg of adsorbents in 10 mL).

Adsorption studies were carried out using 20 mg of adsorbents in contact with 10 batches containing 10 mL of different concentration of CV aquatic solution (from 10 to 100 ppm). The batches were sealed and placed in a shaker for 30 min at 15 °C and pH = 6.5 to obtain the measurement of the adsorption capacity. The experiments were performed in duplicate and average values were reported.

The adsorption capacity was calculated by using Equation (1):

(1) $$\;q_{e}=\frac{\left(C_{0}-C_{e}\right)\times V}{W}$$

where *C*_0_ (mg/L) is the initial dye concentration and *C_e_* (mg/L) is the concentration of dye at equilibrium, *V* (L) is the volume of dye concentration, *W (g)* is the mass of the adsorbent, and *q_e_* (mg/g) is the amount of dye CV adsorbed. The percentage removal of CV was calculated from the formulae given below:

(2) $$R\%=\frac{\left(C_{0}-C_{e}\right)}{C_{0}}\times 100$$

Freundlich and Langmuir models were applied to the experimental data.

##### Freundlich Model

The Freundlich model considers an adsorption taking place on a heterogeneous surface. The isotherm model can be represented by the following equation:

(3) $$lnq_{e}=lnK_{F}+\left(\frac{1}{n}\right)lnC_{e}$$

where *C_e_* is the adsorbate equilibrium concentration expressed in mg/L, $q_{e}$ is the amount of adsorbate in the adsorbent at equilibrium expressed in mg/g, *K_F_* is the Freundlich constant representing the affinity of the adsorptive towards the adsorbing material, 1/n is the Freundlich constant, representing the degree of affinity adsorptive/adsorbing material and indicating how much the adsorption process is favored (*n* < 1 indicates a poor adsorption and the desorption as the favored process, 1 < *n* < 2 indicates a good equilibrium between adsorption/desorption, 2 < *n* < 10 represents a very good adsorption going towards an irreversible phenomenon [55]).

*K_F_* and n can be determined from the linearized plot ln *q_e_* vs. *C_e_* (not reported for the sake of brevity).

##### Langmuir model

The Langmuir model assumes the adsorption reaches a monolayer of coverage [56].

The general equation is

(4) $$q_{e}=\frac{q_{o}bC_{e}}{1+bC_{e}}$$

where $C_{e}$ is the adsorbate equilibrium concentration expressed in mg/L, $C_{e}$ is the adsorbate initial concentration expressed in mg/L, $q_{e}$ is the amount of adsorbate in the adsorbent at equilibrium expressed in mg/g, $q_{o}$ is the monolayer coverage capacities expressed in mg/g, $K_{L}$ is the Langmuir constant indicating the ratio of reagents and products at the equilibrium.

In the linearized form Equation (4) becomes

$$\frac{C_{e}}{q_{e}}=\;\frac{1}{K_{L}q_{o}}+\frac{C_{e}}{q_{o}}$$

or

$$\frac{1}{q_{e}}=\frac{1}{q_{o}}+\frac{1}{K_{L}q_{o}C_{e}}$$

The plot reporting $\frac{1}{q_{e}}\;\mathrm{vs}\;\frac{1}{C_{e}}$ allows to obtain q_0_ and K_L_.

The value,

(5) $$R_{L}=\frac{1}{1+K_{L}C_{0}}$$

indicates if the adsorption is unfavored (if $R_{L}>1$), if it shows a linear trend (if $R_{L}=1$) or if the adsorption is favored (if $0<R_{L}<1$). $\;R_{L}=0$ indicates that the reaction is irreversible.

#### 2.4.4. Contaminants of Emerging Concern (CECs) Adsorption Study

Kinetic studies were performed using 10 ppm of Atenolol and Carbamazepine in contact with 20 mg of **A**-BBS0.4 in a volume of 10 mL, at 20 °C and pH 6.5. Adsorption studies were performed using different amounts of Atenolol and Carbamazepine in contact with 20 mg of **A**-BBS0.4 in duplicate. The mixtures were kept under stirring at 20 °C, pH 6.5 and the residual amount of contaminants was measured at 30 min-contact time in order to obtain the adsorption isotherms.

## 3. Results and Discussion

### 3.1. Materials Characterization

#### 3.1.1. FTIR Spectroscopy

**Figure 1:**
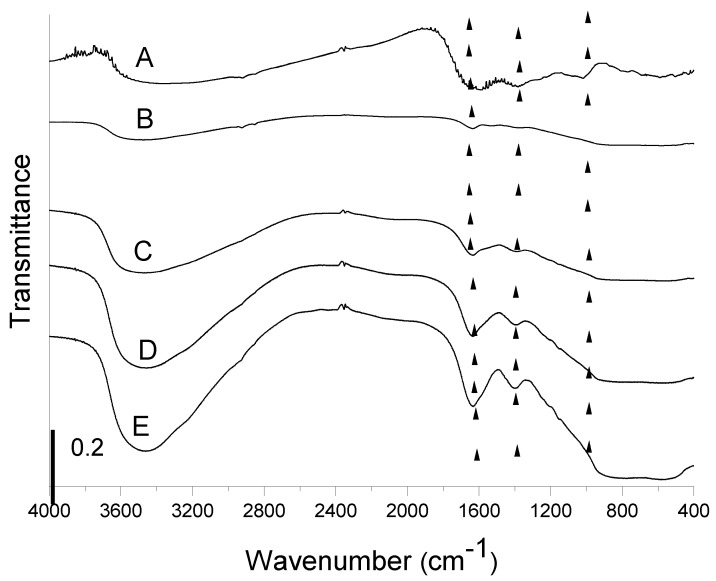
FTIR spectra of Bio-Based Substances (BBS) (curve **A**) pure gamma alumina (**B**), **A**-BBS0.1 (**C**), **A**-BBS0.2 (**D**), and **A**-BBS0.4 (**E**). The spectra were shifted for the sake of clarity, the vertical lines represent the most intense signals due to BBS.

The IR spectra of alumina before and after BBS immobilization were collected in order to assure BBS presence on hybrid alumina systems. The recorded spectra, together with the reference BBS spectrum, are reported in Figure 1. The pure alumina sample shows the presence of atmospheric water molecules interacting with the surface and producing a signal at 1630 cm^−1^ (δ_HOH_ vibration) and a large absorption around 3500 cm^−1^ (ν_OH_ vibrations), whereas the intense signal at low wavenumbers (<1000 cm^−1^) is due to alumina framework vibrations. Pure BBS shows again an intense signal at around 3500 cm^−1^ due to OH groups and atmospheric moisture interacting with the surface (ν_OH_ vibrations), a large signal at 1600 cm^−1^ due to both carbonyl (C=O) stretching and vibration of water molecules adsorbed at the surface (δ_HOH_ signal), and other two signals at 1400 and 1000 cm^−1^ due to carboxylic acid/C–H bending and OCO vibrations, respectively. Another very weak signal can be observed at around 3000 cm^−1^ due to _CH_ stretching vibrations.

Specific signals due to BBS (evidenced in the Figure by vertical lines) are visible in the spectra of the hybrid materials at around 1600, 1400, and 1000 cm^−1^, whereas the less intense signals at around 3000 are not visible due to the small amount of BBS immobilized on the support. In any case, the presence of these absorptions confirms that alumina functionalization was carried out and, moreover, their increasing intensity suggests an increase of the amount of the functionalizing phase on the **A** support increasing the amount of BBS used during the material preparation. No modification of the signal positions was observed with respect to pure BBS (see curve A in the Figure), as expected for a simple electrostatic interaction occurring between alumina surface and BBS molecules. Moreover, it is possible to evidence an important contribute of the hydration layer on the hybrid samples surface, as witnessed by the growth of the signal at 1600 (δ_HOH_ vibration) and 3500 cm^−1^ (ν_OH_), given the polar nature of BBS molecules leading to a stronger interaction with atmospheric moisture.

#### 3.1.2. Gas-Volumetric N2 Adsorption at 77K

**Figure 2:**
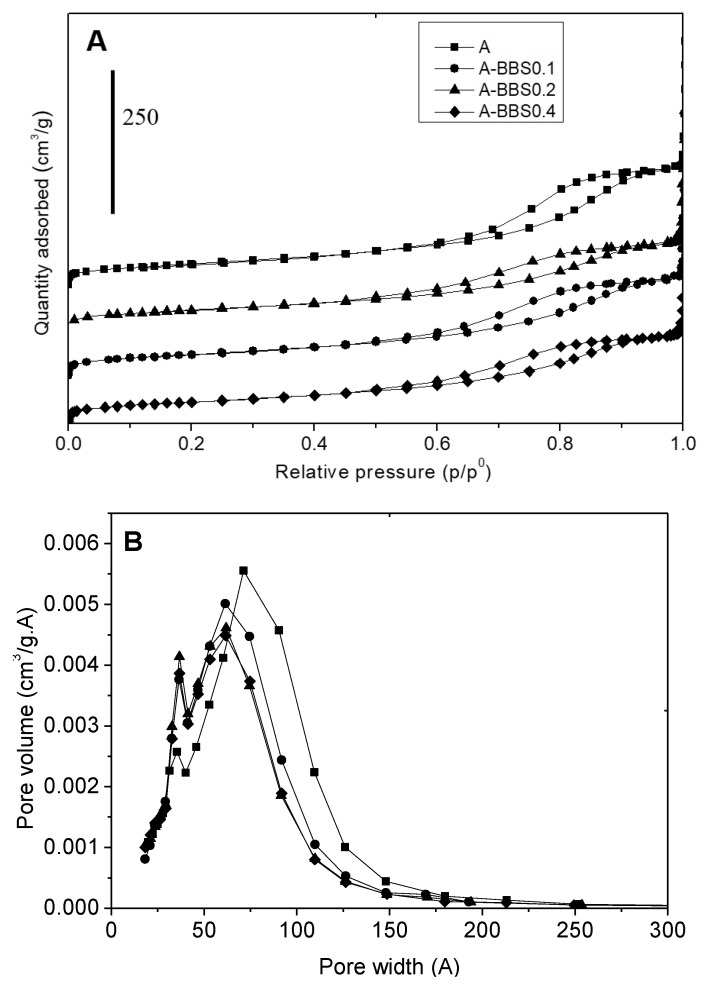
Nitrogen adsorption-desorption isotherms (section **A**) and BJH desorption pore size distribution (section **B**) of reference alumina (squares) and hybrid materials: **A**-BBS0.1 (circles), **A**-BBS0.2 (triangles), and **A**-BBS0.4 (diamond). In section **A** the curves were shifted for the sake of clarity.

The adsorption/desorption isotherms of all hybrid materials and reference non-modified sample are of the IV type (IUPAC classification) indicating that all materials are mesoporous (Figure 2A).

As it can be seen, the hysteresis loops of all the samples are very similar, although the height of the step decreases by increasing the amount of BBS, indicating a minor amount of mesopores after functionalization of the support. Nevertheless, the amount of BBS immobilized seems to be not enough to modify the featuring morphology of the material. The values of BET specific surface area of the materials are reported in Table 2 together with the total amount of mesopores, evaluated by BJH model on the desorption branch of the isotherms, whereas the pore size distribution curves are reported in Figure 2B. The specific surface area of the samples decreases increasing the amount of BBS, although to a very limited extent. This suggests that BBS is not immobilized at the external surface of the particles, since they do not act as aggregating agent between the single alumina particles, as the loss of specific surface area of the material after functionalization should be much higher. This indicates that BBS are allocated essentially into the mesopore empty space, probably because in that space the electrostatic interaction of the support with the molecule can be stronger. The pore size distribution curves allow obtaining other important details concerning the BBS–solid interaction. All samples show two families of mesopores, the first one quite narrow centered at 37 Å of width and another one very wide covering the values 20–180 Å of width. Both families of pores are affected by support functionalization because the large mesopores decrease in amount and width, the relative maximum shifting from 80 to 60 Å of width, whereas the smaller ones increase in intensity after functionalization. This feature suggests that the smallest pores are not involved in the functionalization, probably because of the limited dimensions, whereas BBS are placed into the largest pores, which are those decreasing in amount and size after functionalization causing the increase of the amount of the smallest ones. Also the fact that the hystheresis loops of the isotherms do not change shape after functionalization confirms again that BBS molecules enter into the pores sticking at the internal walls of the solid (causing a decrease of their size) rather than fixing at their entrance (causing the modification of the pore shape which is not observed indeed).

#### 3.1.3. TGA

**Figure 3:**
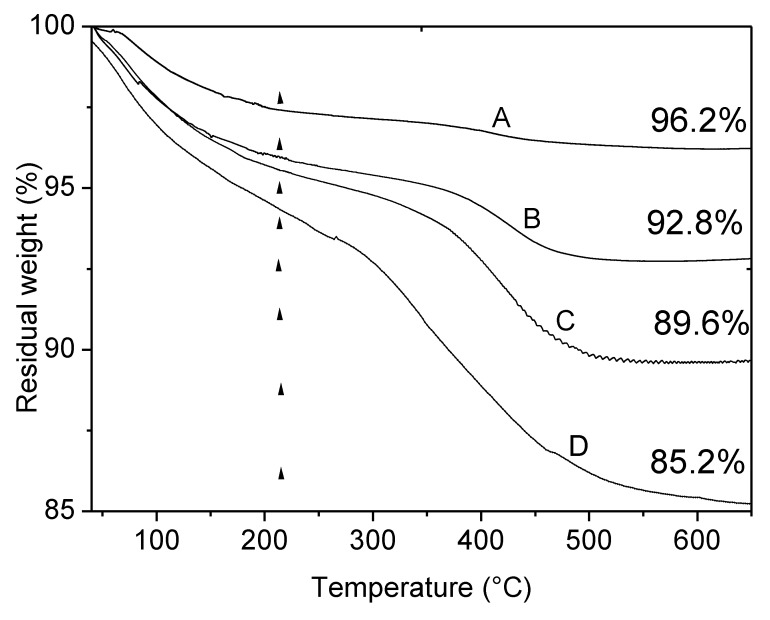

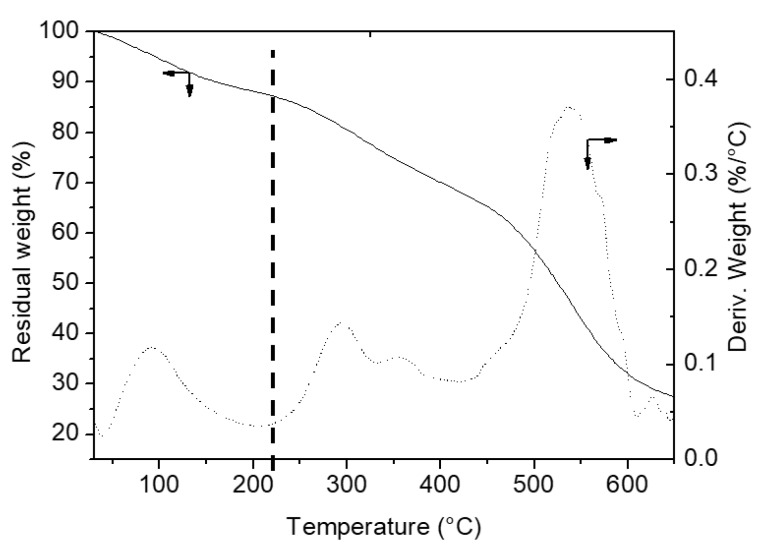
Thermogravimetric analysis (TGA) curves obtained in air. **Upper section**: **A** (curve **A**), **A**-BBS0.1 (**B**), **A**-BBS0.2 (**C**) and **A**-BBS0.4 (**D**); **lower section**: the residual weight (solid line) and derivative residual weight (dotted line) of BBS. The values indicated in the upper picture indicate the residual weight measured for the materials, the vertical dotted lines indicate the temperature of 220 °C used for the quantification of water and organics loss.

Figure 3, upper section, shows the results of TGA analysis on sample **A** before and after immobilization of BBS in the temperature range 40–650 °C under air, Figure 3, lower section, reports the curve related to BBS residual weight registered in the same conditions. As reported in the literature [57], BBS evidences two main regions of weight loss, the first in the range 40–220 °C related to physisorbed and chemisorbed water elimination, the second, occurring in two steps, in the range 220–650 °C related to the oxidation of BBS. Analogously, all the curves of **A** and hybrid materials present two important weight losses. The foster falls in the range 40–220 °C and is due to the removal of adsorbed water molecules: this contribution increases increasing the amount of BBS on the hybrid samples as a consequence of the increased interaction with atmospheric moisture given by the increased polarity of the materials. The latter falls in the range 220–650 °C and deserves some comments. Alumina sample possesses surface OH groups that can be partially eliminated via condensation reaction in form of water molecules at high temperature: the weight loss observed in the range 220–650 °C (1.1%) can be assessed to this phenomenon. Otherwise, the hybrid materials treated at high temperature can experience both the OH elimination via condensation reaction and the elimination of organic moieties through an oxidation reaction which forms CO_2_ and H_2_O. From the comparison of the four curves, it is possible to quantify the amount of the three contributions, namely adsorbed water molecules, amount of OH eliminated via condensation reaction and organic content, for the four samples as reported in Table 3.

The amount of BBS actually loaded onto the support is obtained by calculating the percentage of weight loss for **A**-BBS0.1, **A**-BBS0.2, and **A**-BBS0.4 in the range of 220 to 650 °C and subtracting that observed for **A** support in the same range. The amount of organic matter actually immobilized on hybrid samples depends on the concentration of the BBS solution used for the functionalization: in these experiments the maximum loading has been reached by **A**-BBS0.4 with 7.2% of organic matter immobilized.

#### 3.1.4. Zeta Potential

The zeta potential measurements have been used in order to evaluate the surface charge of the support and its modification after BBS functionalization. This is a very important indication, dealing with adsorption, in order to forecast the type of substrate that can be subjected to efficient interaction with the materials.

**Figure 4:**
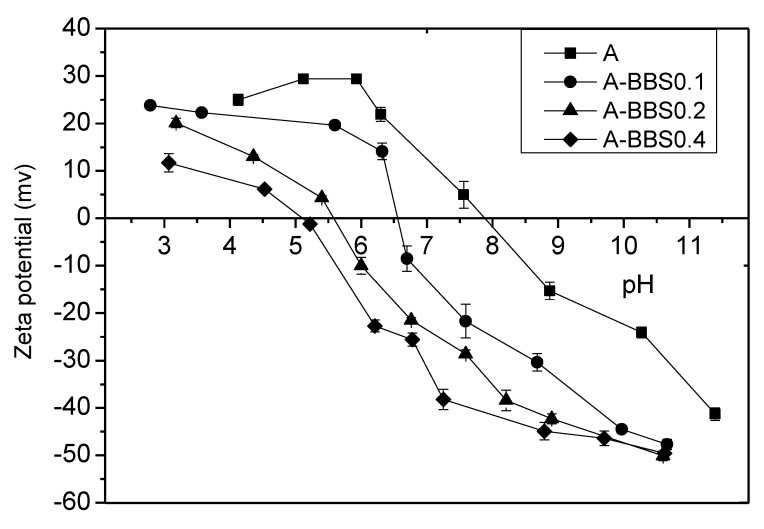
Zeta potential trends of **A** (squares), **A**-BBS0.1 (circles), **A**-BBS0.2 (triangles), and **A**-BBS0.4 (diamonds) as a function of pH.

Figure 4 shows the trend of zeta potential in the pH range 3–11. From the measured trends it is possible to conclude that reference **A** material possesses a positive surface charge in the range 4–7.9 and this makes easy an interaction of the solid with negatively charged substrates. At neutral pH, in particular, BBS molecules bring a negative charge given essentially by dissociated COOH and phenolic Ph-OH groups, therefore the interaction with the alumina positive solid surface occurs very easily. Consequently, the isoelectric point (IEP) of hybrid materials shifts to lower pH increasing the BBS loading.

### 3.2. Removal of Crystal Violet

#### 3.2.1. Effect of CV Concentration

**Scheme 1:**
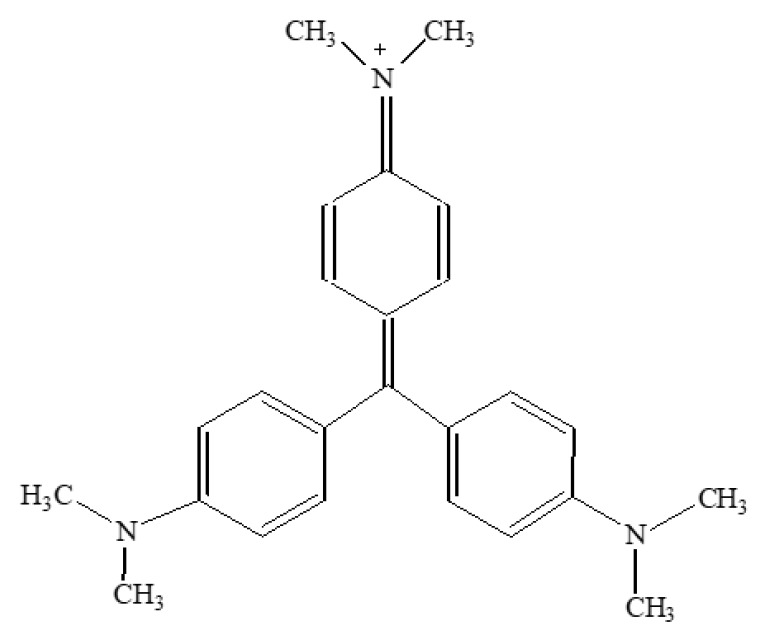
Structure of Crystal Violet.

The chemical structure of CV is reported in Scheme 1.

**Figure 5:**
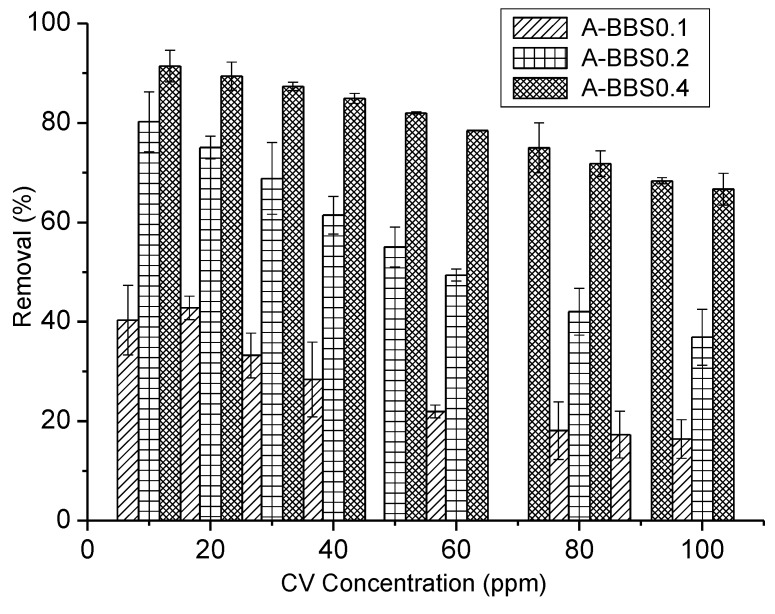
Adsorption % of dye CV by different hybrid adsorbents **A**-BBS0.4, **A**-BBS0.2, and **A**-BBS0.1, versus initial dye concentration at 15 °C.

Different concentrations of dye from 10 to 100 ppm have been used for adsorption experiment keeping constant the amount of hybrid absorbents. Results reported in Figure 5 indicate that an increase of the dye concentration leads to a decrease in the amount of dye adsorbed from 43% to 17% for **A**-BBS0.1, from 80% to 37% for **A**-BBS0.2, and from 91% to 67% for **A**-BBS0.4. Although the removal decreases increasing CV concentration because of the adsorbing materials saturation, the amount of dye removed by **A**-BBS0.4 remains at the value of about 70%, indicating a very good performance. On this material the adsorption of atenolol and carbamazepine was performed.

#### 3.2.2. Adsorption Isotherms and Model Application

**Figure 6:**
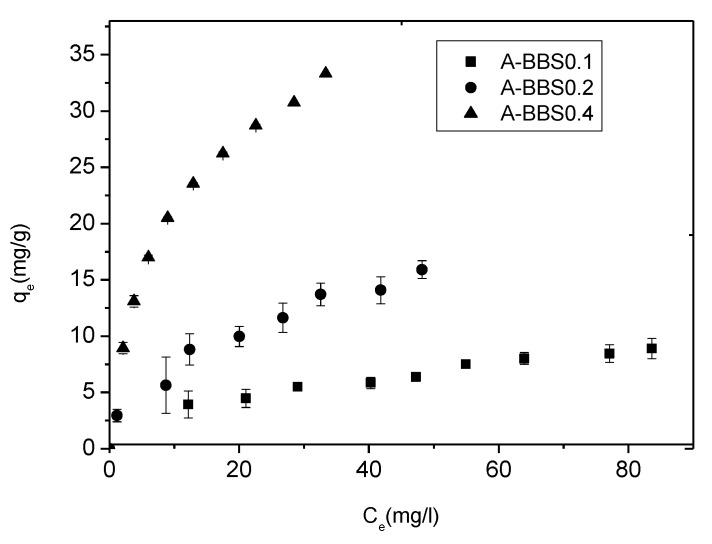
Equilibrium isotherms for the adsorption of CV by different hybrid adsorbents in MilliQ water at 15 °C.

In Figure 6 the removal of CV by the hybrid adsorbents was reported in form of adsorption isotherms. Clearly **A**-BBS0.1 reached the saturation in our experimental conditions, whereas **A**-BBS0.2 and, even more, **A**-BBS0.4 are still far from the saturation.

Langmuir and Freundlich models were applied to the experimental data and the most significant results are reported in Table 4. A very good fitting was obtained applying Langmuir model, as indicated by very high values of r^2^. *q_m_* obtained by this model defines the amount of CV saturating the samples (monolayer amount): the value obtained for the best adsorbent **A**-BBS0.4 corresponds to a maximum adsorption of about 35 mg/g. The R_L_ values (always 0 < *R_L_* < 1) obtained for all the samples indicate a favored adsorption, as also suggested by Freundlich *n* values always higher than 2.

## 4. Removal of CECs

**Scheme 2:**
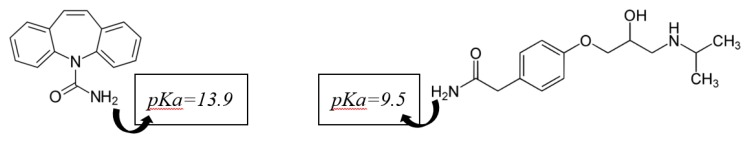
Structure of Carbamazepine [59] (**left side**) and Atenolol (**right side**) and related pKa [60,61].

**Figure 7:**
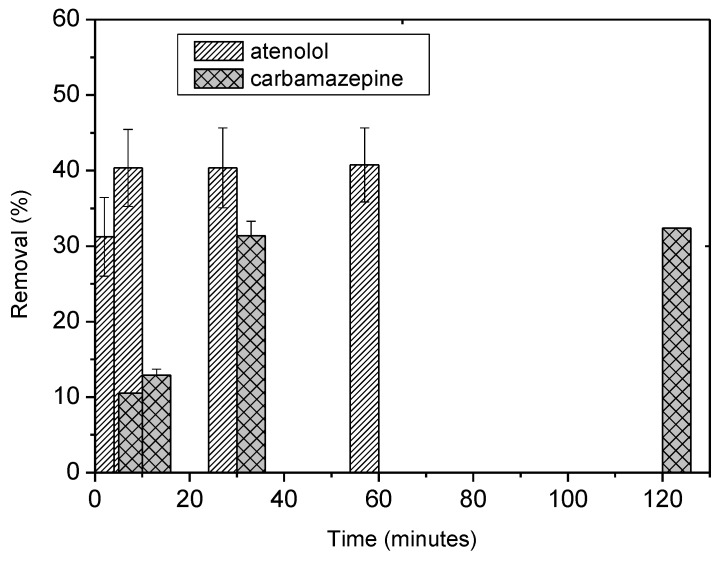
Kinetic of removal of 10 ppm of Carbamazepine and Atenolol at 20 °C by 20 mg of **A**-BBS0.4 in 10 mL of solution expressed in removal percentage.

Figure 7 reports the kinetic of adsorption relative to two different CECs, Carbamazepine and Atenolol, whose structures are shown in Scheme 2. These CECs were chosen on the basis of their chemical structure: both molecules show positively charged structure at pH 6.5 [58], but while Atenolol possessed a branched aliphatic chain, Carbamazepine is a polyaromatic and more compact molecule. 

The efficiency of the material remained acceptable with the larger polar molecule atenolol which is more easily adsorbed with 51% of removal in 30 min thanks to its polar structure and consequently stronger interaction with adsorbent, whereas in the case of carbamazepine only 32% of removal was reached in 30 min suggesting a worse affinity between the material and the polyaromatic substrate.

Langmuir and Freundlich models were also applied to CEC adsorption and the most significant results are reported in Table 5. Good fittings were observed applying both Langmuir and Freundlich models as indicated by very good values of r^2^. *q_m_* obtained by these models defines the amount of Carbamazepine and Atenolol saturating the samples (monolayer amount): the values obtained for the Carbamazepine and Atenolol correspond to maximum adsorption of about 3.06 and 3.26 mg/g respectively. A comparison study of capacity of adsorption for different adsorbents was performed and the results are reported in Table 6. Considering the literature, we have reached promising results in particular in the adsorption of Carbamazepine.

## 5. Conclusions

New hybrid absorbents were prepared following a very easy procedure and characterized via TGA, XRD, Zeta potential, FTIR spectroscopy, and nitrogen adsorption then, they were tested towards the removal of selected pollutants. BBS can functionalize the alumina surface given the electrostatic attraction of opposite charges carried by the two components. In this way, cationic pollutants can be captured by the negatively charged surface of the adsorbent. The electrostatic interaction is, therefore, the major driving force for the adsorption process, but the polarity of the pollutant molecules affects both kinetics and adsorbed amounts as less polar molecules interact at lower extent and in longer time.

**Figure 8:**
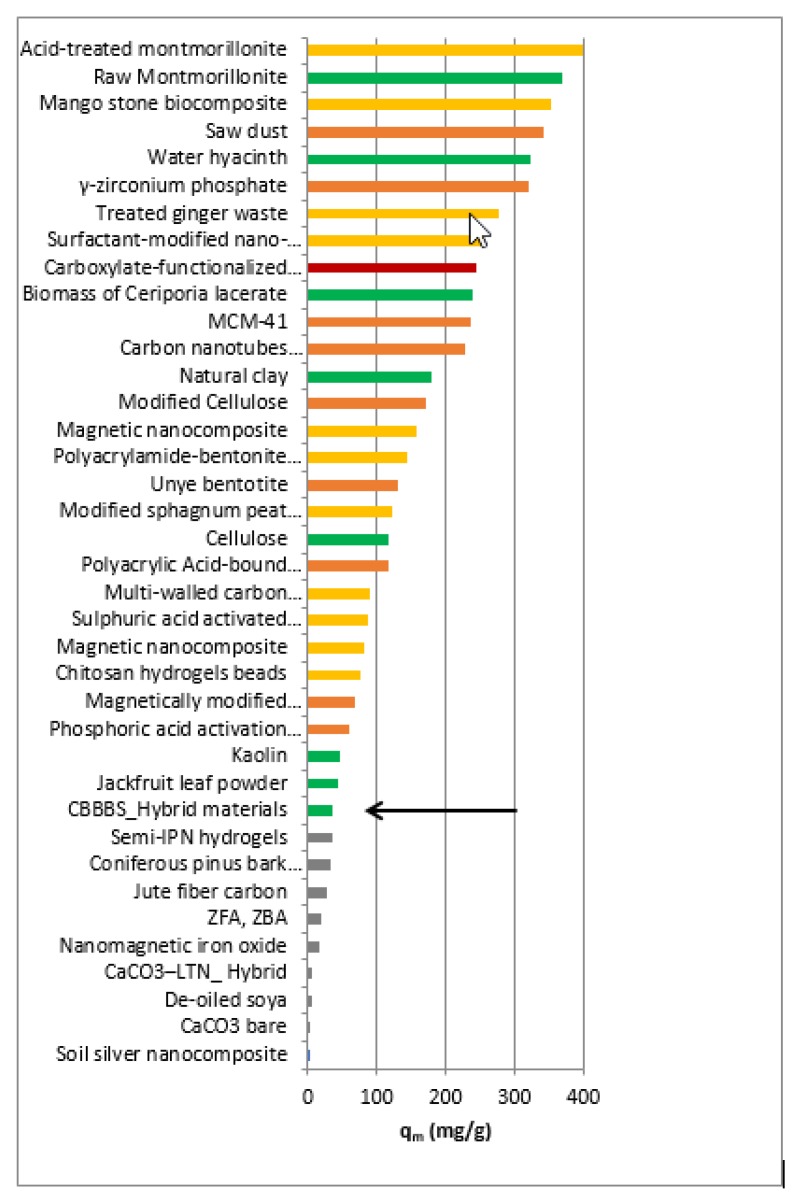
Removal of CV from aqueous solution by **A**-BBS and adsorbents materials reported in the literature and listed in this work (Table 1). The colors have been assigned on the base of aspects related to possible upscaling of the processes involving the materials: energy consumption, multistep/complex synthesis, use of non-green compounds, as described in the text.

The strength of these new hybrid materials is not so much in photocatalytic efficiency, (the comparison in CV removal is reported in Figure 8), as in the simplicity of preparation and in its definitely “green” way. In fact, not only the efficiency of the materials should be taken in account but also other aspects deserve to be considered, i.e., the easiness and the green aspects related to the materials preparation procedure and the energy and economic savings. In fact, several materials with very good performances reported in Table 1 are prepared following complex procedures and use of non-green processes. In order to perform this comparison, we considered only the materials with better performance in CV adsorption with respect to the **A**-BBS hybrid system and we classified them on the basis of the following aspects: energy consumption (calcination and/or thermal treatment at temperature higher than 200 °C), multistep/complex synthesis, use of non-green compounds (acids, bases, solvents and so on). In the classification we assigned a color code from green to orange to red considering the absence of these negative aspects or their simultaneous presence during the preparation. As evidenced in Figure 8, several materials possessing much better adsorption capacity with respect to the **A**-BBS system show an orange or red color, whereas only few, as **A**-BBS0.4, show the green color.

From this perspective, the **A**-BBS materials can be developed further and their performance optimized in view of their use in in-field applications.

## Figures and Tables

**Table 1:** Removal of Crystal Violet (CV) from aqueous solutions by several adsorbents reported in the literature.

Adsorbents	q_m_ (Max Capacity) (mg/g)	Ref.	Temperature °C	pH
Zeolite from fly ash	19.6	[29]	25	5
Acid treated zeolite	17.7	[29]	25	5
Magnetic nanocomposite	158.73	[6]	50	6.5
Soil silver nanocomposite	1.923	[30]	30	4.68
Jute fiber carbon	27.99	[31]	30	9
Semi-IPN hydrogels	35.09	[7]	25	7.4
Coniferous pinus bark powder (CPBP)	32.78	[32]	30	8
Chitosan hydrogels beads	76.9	[33]	30	7
Phosphoric acid activation carbon (PAAC)	60.42	[34]	28	6
Sulphuric acid activated carbon (SAAC)	85.84	[34]	28	6.04
Magnetically modified activated carbon	67.1	[35]	20	9
Nanomagnetic iron oxide	16.5	[35]	20–40	9
Magnetic nanocomposite	81.7	[36]	(10–50)	8.5
Unye bentonite	131	[37]	22	6.5
MCM-41	236.64	[38]	25	4
Saw dust	341	[39]	(15–50)	neutral pH
Modified sphagnum peat moss	121.95	[40]	20	6.5
Polyacrylic Acid-bound magnetic nano particles	116	[41]	25	6
Acid-treated montmorillonite	400.0	[42]	30	5.9
Treated ginger waste	277.7	[43]	(30–50)	6.2
Carboxylate-functionalized cellulose nanocrystals	243.9	[44]	30	6
Mango stone biocomposite	352.79	[45]	33	8
Multi-walled carbon nanotubes	90.52	[46]	25	6–8
Carbon nanotubes supported nanocables	228.30	[47]	(25–45)	9
Polyacrylamide-bentonite composite	144.60	[48]	30	6
γ-zirconium phosphate	320.20*	[49]	25	9
Modified Cellulose	169.9–218.8	[50]	50	9
Surfactant-modified nano-alumina	254.3	[51]	25	4
CaCO3–LTN Hybrids	6.34 *	[52]	25	6
CaCO3 bare	3.35	[52]	25	6
De-oiled soya	5	[16]	30	8

* The related amounts are not q_m_ but refer to the following experimental conditions: In Ref. [49]: 500 mg of adsorbent in 523.3 mL of 1.5 × 10^−2^ M dye in water for a contact time of 48 h, Ref. [52]: 30 mg of adsorbent was poured in 5 mL of 0.1 mM of dye under stirring for 420 min.

**Table 2:** Texture features for reference and hybrid materials.

Materials	BET Specific Surface Area (m^2^/g)	BJH Pore Volume (cm^3^/g)
**A**	186	0.42
**A**-BBS0.1	181	0.33
**A**-BBS0.2	172	0.30
**A**-BBS0.4	165	0.29

**Table 3:** Weight losses observed for reference and hybrid materials.

Materials	40–220 °C (Adsorbed Water %)	220–650 °C (Organic Content and OH Groups Eliminated %)	OH Groups Eliminated (%)	Measured Organic Content (%) ± 0.1
**A**	2.8	-	1.1	-
**A**-BBS0.1	4.4	2.7	1.1	1.6
**A**-BBS0.2	4.9	5.4	1.1	4.3
**A**-BBS0.4	6.5	8.2	1.1	7.1

**Table 4:** Langmuir and Freundlich model parameters calculated by linear fitting of the adsorption data.

Model		A-BBS0.1		A-BBS0.2		A-BBS0.4	
		Value	S.E.	Value	S.E.	Value	S.E
Langmuir	*q_m_* (mg/g)	8.92	0.59	21.38	2.50	35.09	0.61
	*K_L_* (mg/L)	0.065	0.020	0.087	0.048	0.09	0.090
	*r* ^2^	0.975	-	0.977	-	0.992	-
	*R_L_*	0.898		0.861		0.763	
Freundlich	*K_F_* (mg/g) (L/mg)	14.08	5.58	28.547	6.75	67.54	8.455
	*n*	2.52	0.65	2.170	0.203	2.084	0.164
	*r* ^2^	0.995	-	0.94	-	0.978	-

**Table 5:** *q_m_* of adsorption of Contaminants of Emerging Concern (CECs) by **A**-BBS0.4.

Model		Carbamazepine		Atenolol	
		Value	S.E.	Value	S.E.
*Langmuir*	*q_m_* (mg/g)	3.0628	0.06	3.26	0.145
	*K_L_* (mg/L)	0.119	0.057	0.11	0.008
	*r* ^2^	0.917	-	0.904	-
*Freundlich*	*K_F_* (mg/g) (L/mg)	0.405	0.07	4.43	0.13
	*n*	1.526	0.35	0.77	0.006
	*r* ^2^	0.905	-	0.906	-

**Table 6:** Removal of organic pollutants from wastewater.

Adsorbents	*q_m_* (mg/g)	Ref.	CEC	Temperature (°C)	pH
A-BBS0.4	3.26 3.06	This study	Atenolol Carbamazepine	20	6.5
Agricultural soils	0.01	[62]	Carbamazepine	25	9.2
Functionalized silica	0.04	[63]	Carbamazepine	25	7
Granulated cork	1.84	[64]	Carbamazepine	20±2	4.6
Activated carbon	18.8	[65]	Atenolol	25	6
Graphene oxide	95	[66]	Atenolol	25	2
